# Temperature dependent stereodynamics in surface scattering measured through subtle changes in the molecular wave function†

**DOI:** 10.1039/d4fd00007b

**Published:** 2024-02-14

**Authors:** Helen Chadwick, Gil Alexandrowicz

**Affiliations:** a Department of Chemistry, Faculty of Science and Engineering, Swansea University Swansea SA2 8PP UK h.j.chadwick@swansea.ac.uk g.n.alexandrowicz@swansea.ac.uk

## Abstract

A magnetically manipulated molecular beam technique is used to change the rotational orientation of H_2_ molecules which collide with a stepped Cu(511) surface and explore how the polarisation dependence of molecules scattering into the specular channel changes as a function of surface temperature. At all temperatures, H_2_ molecules that are rotating like cartwheels are more likely to be scattered into the specular channel than those that are rotating like helicopters. Furthermore, the scattered molecules are more likely to be rotating like cartwheels, regardless of their state before the collision. Increasing the temperature of the Cu(511) surface causes the polarisation effects to become stronger, with the scattering becoming more selective for H_2_ with cartwheel like rotation. Therefore, scattering a molecular beam of H_2_ from a Cu(511) surface and taking the molecules scattered into the specular channel provides a method to create a rotationally polarised beam of H_2_, where the polarisation can be tuned by changing the surface temperature. In contrast, the rotational orientation dependence observed for specular scattering from a flat Cu(111) surface is independent of surface temperature within the same temperature range.

## Abstract 2

Specular scattering of H_2_ from Cu(511) preferentially occurs for cartwheeling molecules and becomes more selective at higher surface temperatures.

## Graphical abstract

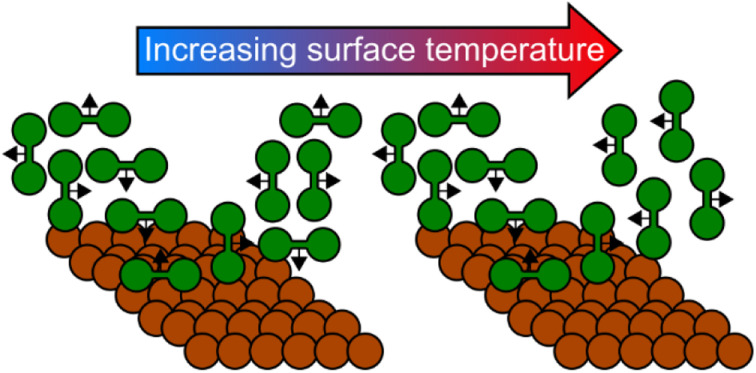

## 1 Introduction

There are many factors that can affect the outcome of a collision between a molecule and a surface, including the translational,^1^ vibrational^2,3^ and rotational^4,5^ energy of the molecule, and the temperature^6^ and plane^7^ of the surface. The rotational orientation of the molecule, which classically corresponds to whether the molecule is rotating like a helicopter or a cartwheel as it approaches the surface, has also been shown to have an effect, with molecules which are rotating like a helicopter being more likely to dissociate than those rotating like a cartwheel.^8,9^ Likewise, there have been a number of studies that show that collisions of molecules with surfaces can create a scattered beam that is rotationally polarised.^10–13^

Theoretical modelling has predicted that surface temperature will influence the rotational orientation dependence of gas–surface reactions, with most work focussing on H_2_ reacting on copper surfaces.^14–17^ These studies have shown that as the surface temperature is increased the dependence of the reaction on the rotational orientation of the molecule is expected to decrease. This can be attributed to both the thermal expansion of the lattice and increased corrugation of the surface due to the thermal motion of the atoms, both of which lower the activation barrier to the reaction.^14–16^ Therefore, the overall reactivity increases and molecules in less favourable geometries can react, both of which contribute to the decrease in the rotational orientation dependence of the reaction at higher surface temperatures.^17^

**Fig. 1:**
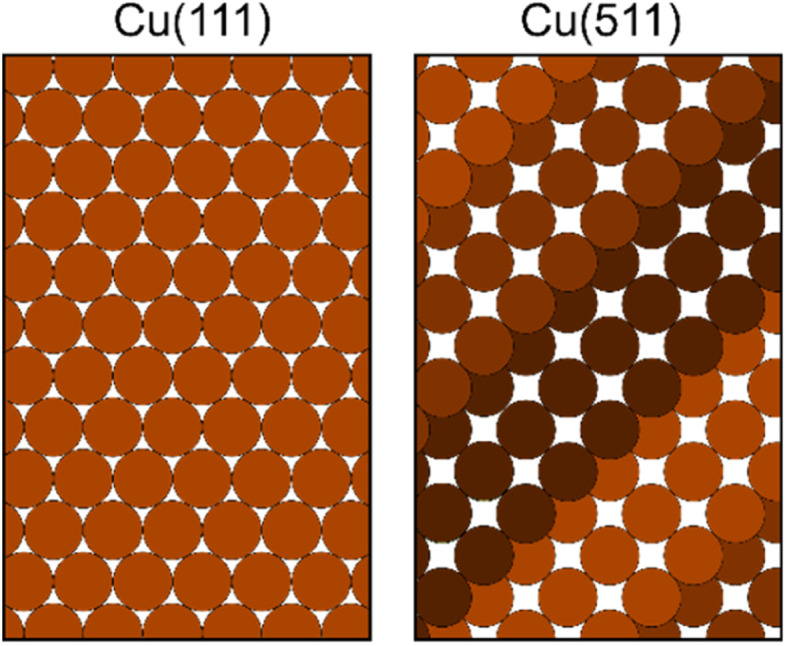
Schematic of the structure of the Cu(111) surface (left panel) and Cu(511) surface (right panel).

Using the magnetic molecular interferometry (MMI) technique,^18^ we have previously shown that elastic scattering is also affected by the initial rotational orientation of the H_2_ in collisions with both LiF^13^ and copper surfaces.^18,19^ Additionally, we have demonstrated that rotationally inelastic scattering can be controlled by changing the initial rotational orientation of D_2_ molecules before they collide with a Cu(111) surface.^20^ The present work builds on these previous studies and makes use of the MMI methodology to compare the effect that rotational orientation has on the elastic scattering of H_2_ (specifically ortho-H_2_) from two different copper surfaces as a function of surface temperature. Ortho-H_2_ molecules have a nuclear spin (*I*) of 1, and a rotational angular momentum (*J*) of 1, which split into 9 non-degenerate *m*_*I*_, *m*_*J*_ (nuclear spin projection and rotational orientation projection) states in the applied magnetic fields in the apparatus, allowing us to control and manipulate these states before they collide with either a Cu(111) or Cu(511) surface. The Cu(111) surface has a flat, hexagonal close packed structure, as shown schematically in the left panel of Fig. 1, whereas Cu(511) has a stepped structure with approximately 3 atom wide (100) terraces separated by (111) steps, as shown schematically in the right hand panel of Fig. 1. This work therefore complements the first study performed with the MMI apparatus which showed that there was a stronger rotational orientation effect for H_2_ scattering from Cu(511) than Cu(111),^18^ by quantifying this effect for scattering from Cu(511) as well as studying how changing the surface temperature changes the polarisation dependence of the scattering.

The rest of the paper is organised as follows. In the next two sections, we briefly describe the MMI experiment followed by the methods we use to analyse the signals that we measure. Then, we present the results and discussion, focussing on how changing the surface temperature affects the polarisation properties of H_2_ scattering from the Cu(511) surface, before summarising the main results and conclusions.

## 2 Experimental methods

The MMI apparatus has been described previously^18–23^ and only the details relevant for the current work are presented here.

**Fig. 2:**
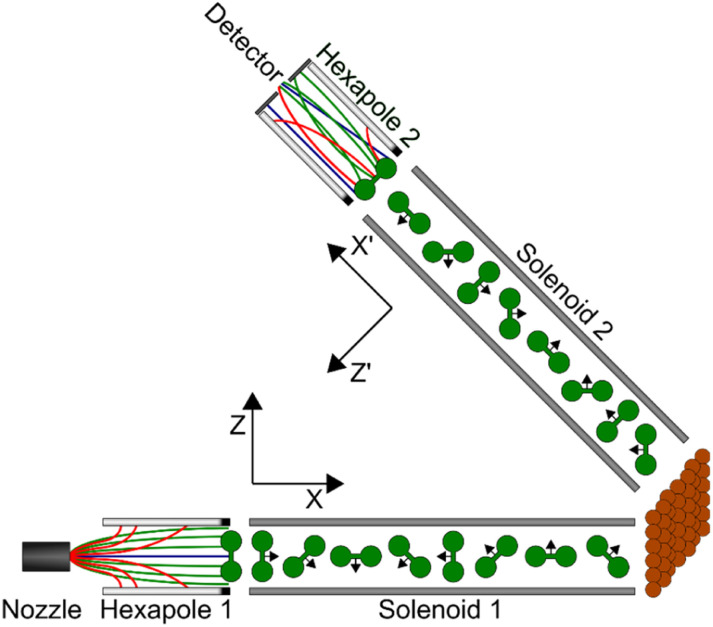
Schematic of the magnetic molecular interferometer apparatus showing the positions in the beamline of the various magnetic elements discussed in the text, as well as the axes used to define the quantisation axes at different points in the propagation.

A continuous molecular beam is generated using a skimmed supersonic expansion of H_2_ through a nozzle held at a temperature of 100 K, which results in a velocity of approximately 1460 m s^−1^ with a full width at half maximum on the order of 6%. This corresponds to a collision energy of approximately 22 meV which allows us to focus on rotationally elastic events as it is insufficient to obtain rotationally inelastic (*J* state changing) scattering just through translational to rotational energy transfer. After two differential pumping stages, the beam enters the first magnetic hexapole,^24^ where due to the strong magnetic field gradient the molecules experience a force depending on their magnetic moment, *i.e.*, on their *m*_*I*_, *m*_*J*_ state. Due to the nuclear magnetic moment being approximately seven times greater than the rotational magnetic moment,^25^ the focussing of the states depends more strongly on the *m*_*I*_ state than the *m*_*J*_, state, with molecules in the *m*_*I*_ = −1 state being focussed to a parallel beam, the *m*_*I*_ = 1 state deflected outwards, and the *m*_*I*_ = 0 state mostly unaffected by the hexapole field. Due to the strong magnetic field gradients within the hexapole lens, the superposition states decohere^26^ with the result that at the end of the hexapole all nine initial (pure) *m*_*I*_, *m*_*J*_ states are still populated, but with unequal populations which are related to the probability that the state is transmitted through the magnetic lens. Immediately after the hexapole, there is a hexapole to dipole transition which adiabatically rotates the magnetic moments so that the projection states are defined with respect to a single quantisation axis, which is denoted *Z* in Fig. 2.

After a zero-field region, the H_2_ molecules enter a solenoid which generates a tuneable magnetic field either anti-parallel or parallel to the *X* axis (for positive and negative currents (and correspondingly fields) respectively). In the measurements presented here, this is scanned between −0.15 A and 0.15 A using a high stability Danfysik power supply which generates a magnetic field in the first arm of the apparatus of between −16.8 and 16.8 gauss metre. Classically, this magnetic field causes the magnetic moments of the H_2_ molecules to undergo Larmor precession, which changes the fraction of the molecules that are rotating like a helicopter compared to those that are rotating like a cartwheel. Quantum mechanically, the field transforms an initially pure *m*_*I*_, *m*_*J*_ state into a coherent superposition state with complex amplitudes that undergo Rabi oscillations during the flight through the field. This state evolution is manifested as oscillations of both the *m*_*I*_, *m*_*J*_ state populations as well as the relative phase of the superposition state. The Cu(511) or Cu(111) surface is mounted on a home-built 6-axis manipulator in an ultrahigh vacuum chamber positioned after the exit of the first solenoid. The temperature is monitored using a T-type thermocouple and was stable to within ±0.1 K of the set surface temperature, with an absolute error estimated as ±1 K throughout the scattering measurements presented here. Both samples were prepared using repeated Ar^+^ sputter–anneal cycles, where an annealing temperature of approximately 800 K was used for both Cu(511) and Cu(111), with both low energy electron diffraction (LEED) and helium atom scattering used to verify the cleanliness and order of the surface before performing the H_2_ scattering experiments.

The measurements were performed under specular scattering conditions, so the surface was positioned such that the incidence angle was the same as the outgoing angle (with respect to the surface normal) which is half of the total angle between the two arms of the machine (total angle = 46.2°). Additionally, the Cu(511) surface was aligned so that scattering occurred along an azimuth perpendicular to the direction of the steps. After scattering from the surface, the H_2_ molecules which undergo specular scattering travel through the second arm of the apparatus. The scattered molecules then pass through a second solenoid, which in the measurements presented here was either turned off or operated with a 0.1 A current (generating a field of 11.2 gauss metre) directed along the −*X*′ axis, which is controlled by a second (independent) Danfysik power supply. After the solenoid, there is a zero field region followed by a dipole to hexapole transition element which defines the quantisation axis as being along *Z*′, before a second hexapole^27^ which again deflects molecules depending on their *m*_*I*_, *m*_*J*_ state. At the end of the beam line there is a highly sensitive custom-built mass spectrometer detector.^28^

## 3 Data analysis

The methods that are used to analyse the MMI data have also been described previously^13,20^ and so are only briefly summarised here. To be able to analyse the data from the experiments, it is necessary to model how each of the *m*_*I*_, *m*_*J*_ states of the H_2_ molecule interacts with the applied magnetic fields along the beam line of the apparatus. In our previous work,^13,19,20,22^ we have used semi-classical trajectory calculations^29^ to determine the probability that the different states are transmitted through the first and second magnetic hexapoles separately, but here we have improved the model to account for the trajectory of the molecules through both arms of the machine, *i.e.*, the probability of a molecule being transmitted through the second hexapole depends on the state it was in when transmitted through the first due to small but non-negligible changes in the angular and spatial distribution of the beam at the entrance to the second hexapole. As will be shown in a future publication, this modification improves the fits that are obtained to the data but does not have any effect on the conclusions on the stereodynamic properties that are presented here. We denote the probability that has been calculated for the transmission of an initial state *n* through the first hexapole, and final state *f* through the second as *P*_hex_(*f*,*n*).

The coherent evolution of the nine initial *m*_*I*_, *m*_*J*_ states through the first and second arms of the apparatus is calculated using a mixed classical–quantum approach which has been described previously,^18^ with the motion of the centre of mass of the molecule treated classically but the evolution of the wave function describing the initial *m*_*I*_, *m*_*J*_ state propagated quantum mechanically through the measured 3-dimensional magnetic field profile of the apparatus using the Hamiltonian given in ref. 25. This is performed separately for both arms of the apparatus which produces the propagation matrices *U*(*B*_1_) and *U*(*B*_2_), which characterise the coherent superposition state that an initially pure *m*_*I*_, *m*_*J*_ state becomes due to passing through the first and second arm of the machine, respectively.

The change of the quantum states defined with respect to the surface normal when the molecules scatter from the surface is characterised using a scattering-matrix (*S*). Using the surface normal as the quantisation axis could be considered to be an arbitrary choice, as the *S*-matrix with respect to a different quantisation axis could be determined by multiplying the *S*-matrix with respect to the surface normal by the appropriate rotation-matrix (or matrices). The surface normal was chosen here to be consistent with the convention used in previous theoretical studies of molecule–surface scattering.^30^ Strictly speaking a 9 × 9 matrix is needed to describe the collision process. However, since the spin–rotation coupling has a negligible effect during the short interaction time and since the copper surfaces used in the present study are non-magnetic, *m*_*I*_ is assumed to be unaffected by the collision. This means that we can focus on a 3 × 3 scattering-matrix where each element is a complex number which corresponds to an initial *m*_*J*_ state (*n*′) to final *m*_*J*_ state (*f*′) transition. This reduced dimensionality matrix is then expanded to a 9 × 9 matrix to calculate the wave function after the scattering event. Whilst the 3 × 3 matrix contains nine amplitudes *s*_*f*′*n*′_ and nine phases *k*_*f*′*n*′_ the reflection symmetry of the surface reduces the number of parameters,^20^ because, in effect, the surface cannot distinguish between molecules that are rotating like clockwise and anti-clockwise helicopters. This means that the scattering-matrix for the elastic scattering of H_2_ in *I* = 1, *J* = 1 reduces in complexity even further to1
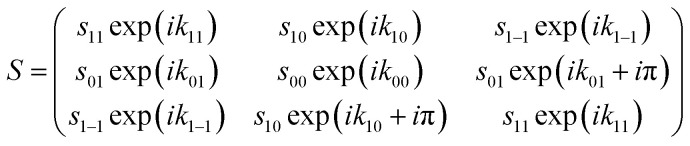
It follows that the coherent superposition state at the start of the second hexapole for a molecule that is in an initial pure state |*n*〉 at the end of the first hexapole can be calculated as^13,18,20^

2 |*Ψ*_*n*_〉 = *U*(*B*_2_)*R*(*θ*_2_)*SR*(*θ*_1_)*U*(*B*_1_)|*n*〉

where *R*(*θ*_1_) and *R*(*θ*_2_) are rotation matrices which change the quantisation axes from the direction of the dipole in the first arm of the machine (*Z* in Fig. 2) to the surface normal, and from the surface normal to the dipole direction in the second arm of the machine (*Z*′ in Fig. 2) respectively. The signal that is measured in the experiment can then be calculated using^18^3
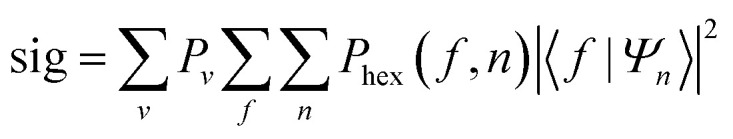
where *P*_*v*_ accounts for the velocity distribution and is modelled as a Gaussian, and the sums run over the velocities present in the molecular beam as well as the nine initial and nine final *m*_*I*_, *m*_*J*_ states.

To extract a scattering-matrix from the data, the signals measured scanning *B*_1_ at *B*_2_ = 0 gauss metre (*i.e.*, with the second solenoid off) and *B*_2_ = 11.2 gauss metre were fit simultaneously using the Nelder and Mead downhill simplex algorithm^31^ in combination with simulated annealing to minimise the difference between the signal calculated with the fit parameters and the experimental data. An initial set of fits were run at each surface temperature to determine the velocity distribution which best characterised the frequency and decay of the oscillations, which was then used in a second set of fits where only the 10 scattering-matrix parameters given in eqn (1) and a background were allowed to vary. This was repeated 100 times for each dataset, with different randomly generated initial parameters used for each fit, which, in combination with the simulated annealing algorithm, helps to ensure that the parameters obtained from the best fits correspond to the global minimum rather than a local minimum.

## 4 Results and discussion

**Fig. 3:**
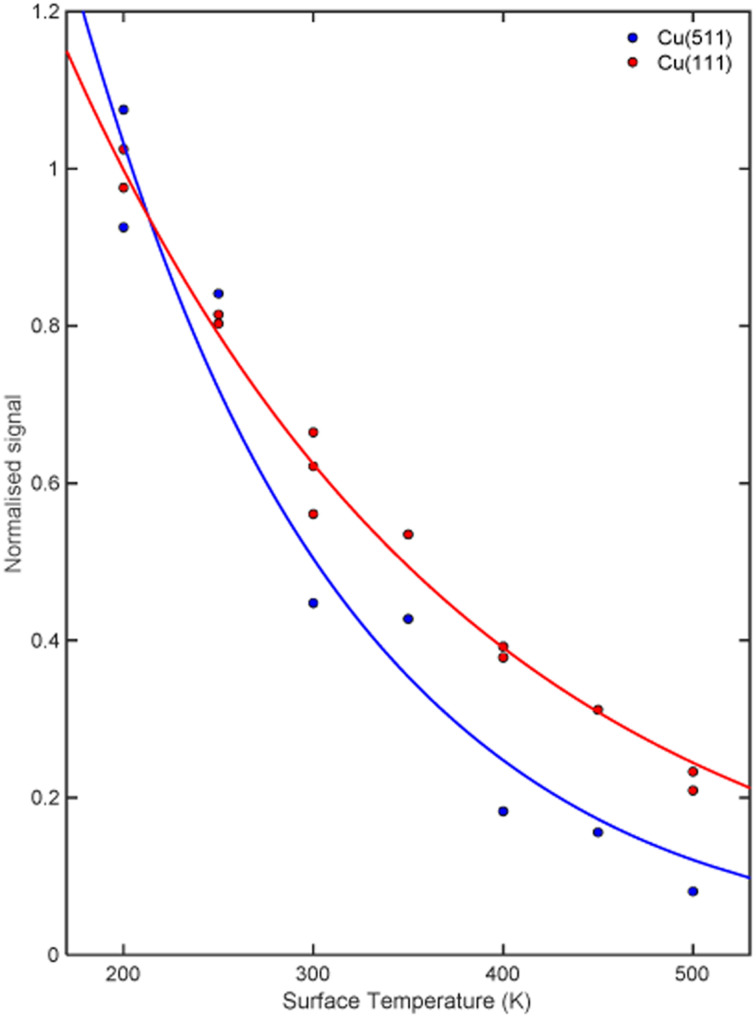
Signal intensity as a function of surface temperature for the specular scattering of H_2_ from a Cu(511) surface (blue) and a Cu(111) surface (red) normalised with respect to the average signal intensity measured at a surface temperature of 200 K. The solid lines are exponential decay fits to the data, to guide the eye.

The signal intensity for the specular scattering of H_2_ from Cu(511) (blue) and Cu(111) (red) as the surface temperature is increased is shown in Fig. 3. Due to the angular resolution of the machine (approximately 0.02°) the only significant contribution to this intensity is from translationally and rotationally elastic (*J* state conserving) collisions, with any inelastic contributions being scattered into a background that is at least 50 times smaller than the elastically scattered signal. To aid the comparison, the signal intensities have been normalised to the average value measured at a surface temperature of 200 K. A reduction in signal which follows an exponential decay (blue and red line for Cu(511) and Cu(111) respectively) as a function of temperature is seen for both surfaces, with the decay being considerably faster for the stepped Cu(511) surface. There are various mechanisms that can lead to a decay in the specular scattering intensity with increasing surface temperature including increased vibrational amplitudes,^32–34^ increased density of surface defects,^35,36^ lattice expansion^37^ and structural changes.^38,39^ These are expected to be different for flat and stepped surfaces,^40^ in particular it is known that Cu(511) undergoes a roughening transition at a temperature of 380 K.^41^ Attempting to interpret these results to identify the dominant mechanism behind this decay is beyond the scope of this work. However, any attempt to do so would require us to consider that the molecules approach the surface with different rotational orientations, and that each of the potential mechanisms mentioned above is likely to depend on the rotational orientation, introducing further ambiguity to an interpretation task which is already very complex. Here we will use the MMI technique to explore how the scattering of the different rotational orientation projection states changes as the surface temperature is increased.

**Fig. 4:**
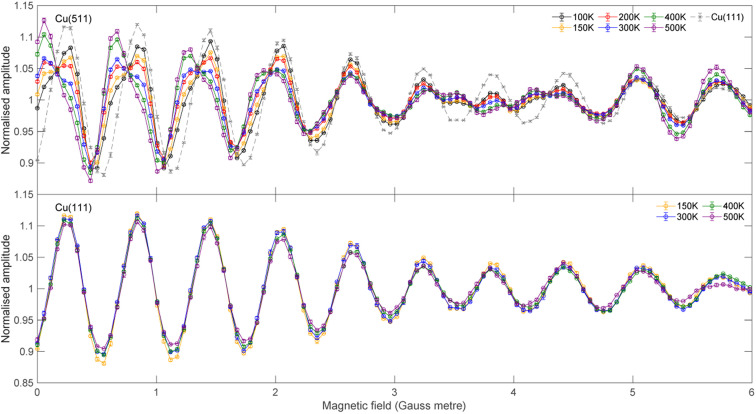
Top panel. MMI oscillation curves measured for H_2_ scattering under specular conditions from Cu(511) at surface temperatures of 100 K (black), 150 K (orange), 200 K (red), 300 K (blue), 400 K (green) and 500 K (purple). For ease of comparison, the equivalent measurement for Cu(111) at a surface temperature of 150 K is shown as a grey dashed line. Bottom panel. Oscillation curves measured for H_2_ scattering under specular conditions from Cu(111) at surface temperatures of 150 K (orange), 300 K (blue), 400 K (green) and 500 K (purple).

MMI oscillation curves measured by scanning *B*_1_ for a fixed value of *B*_2_ = 0 gauss metre for the specular scattering of H_2_ from a Cu(511) surface at different temperatures are shown in the top panel of Fig. 4. For comparison, similar measurements for scattering from a Cu(111) surface are shown in the lower panel. The oscillation curves from the two surfaces are different; to make this easier to see, the Cu(111) MMI signal measured at *T*_S_ = 150 K (grey line) is also presented in the top panel. This difference, which means different stereodynamics of the scattering process, is expected due to the different structures of the surface, with the Cu(111) surface having a flat, hexagonal close packed structure, whereas the Cu(511) surface consists of approximately 3 atom wide (100) terraces separated by (111) steps, as shown schematically in Fig. 1.

A more interesting difference is that while the Cu(111) curves remain very similar when the surface temperature is increased, prominent changes are seen in the curves measured from the stepped Cu(511) surface. This means that the stereodynamics, *i.e.*, the rotational orientation dependence of specular scattering, is temperature dependent. To be able to quantitatively assess the temperature dependence of the stereodynamics of the scattering, we extract the *S*-matrix parameters using the best fit procedure described briefly above and in ref. 13. Obtaining a unique set of *S*-matrix parameters from MMI measurements requires a second complementary measurement at a different *B*_2_ value. As has been shown in the past,^13^ this produces two different interference patterns and unique scattering-matrix parameters can be extracted by simultaneously minimising the fit error on both data sets.

**Fig. 5:**
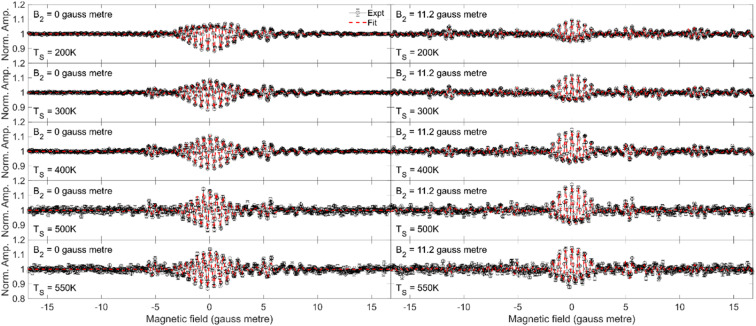
Fits (red dashed line) to the experimental oscillation curves (black) measured for H_2_ scattering from a Cu(511) surface at a temperature of 200 K (first row), 300 K (second row), 400 K (third row), 500 K (fourth row) and 550 K (fifth row) for *B*_2_ = 0 gauss metre (left column) and *B*_2_ = 11.2 gauss metre (right column).

In the current work, we will limit the quantitative analysis to the Cu(511) data, as the Cu(111) data was not taken along a known crystal azimuth. The measurements (black circles) performed for fixed values of *B*_2_ = 0 gauss metre (left column) and *B*_2_ = 11.2 gauss metre (right column) are presented in Fig. 5 for H_2_ scattering from Cu(511) at surface temperatures of 200 K (first row), 300 K (second row), 400 K (third row), 500 K (fourth row) and 550 K (fifth row). The most significant oscillation is observed at *B*_1_ values of around 0 gauss metre. When *B*_2_ = 11.2 gauss metre, these oscillations can be attributed to the different *m*_*J*_ states having different probabilities of scattering from the surface into the specular channel, which changes the number of molecules (or flux) down the second arm of the apparatus,^18,19^ whereas when *B*_2_ = 0 gauss metre, there will additionally be a spin-echo.^19,23,42^

The dashed red lines in each of the panels in Fig. 5 correspond to the best fit to the measured MMI oscillation curve. The fits to the data reproduce all the features that are observed in the measurements, allowing us to extract empirical scattering-matrix parameters, which are presented in the ESI.†

**Fig. 6:**
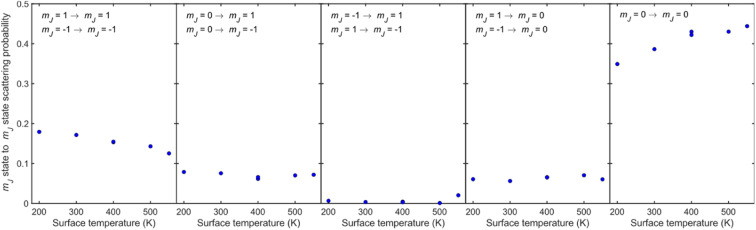
*m*_*J*_ state to *m*_*J*_ state scattering probabilities obtained from the best fits (with lowest errors) as a function of surface temperature. The initial and final *m*_*J*_ state is given in the text of each panel.

From the scattering-matrix amplitudes we can calculate *m*_*J*_ state to *m*_*J*_ state scattering probabilities^43^ as4
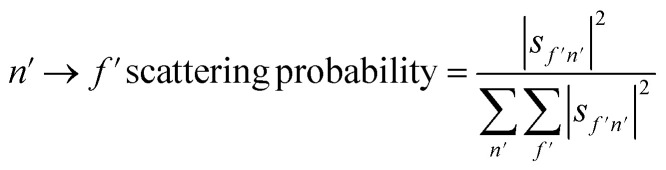
where the quantisation axis corresponds to the surface normal. The state resolved scattering probabilities are presented in Fig. 6, where the label in each panel states the initial and final *m*_*J*_ states the plot corresponds to. The number of independent fits that gave the probabilities shown are given in Table 1, and we demonstrate in the ESI† that increasing the number of fits in the analysis does not affect the trends presented here. There are two data-points at *T*_S_ = 400 K which correspond to values obtained from two different independent MMI measurements, to demonstrate the reproducibility of the data acquisition and the analysis.

**Table 1:** Number of fits (out of 100) that gave the probabilities (rounded to the nearest 0.01) presented in the figures in the paper

Dataset	200 K	300 K	400 K	400 K repeat	500 K	550 K
Number of best fits	21	18	15	18	24	26

For all temperatures, the probabilities for *m*_*J*_ state conserving collisions (first and fifth panels) are larger than those which change the *m*_*J*_ state (second, third and fourth panels), although the probability is lower for helicopter type rotation (first panel) than for cartwheel type rotation (fifth panel). When the *m*_*J*_ state changes, scattering from Cu(511) is more likely to lead to a change in the plane of rotation *i.e.*, molecules that are rotating like helicopters becoming cartwheels and *vice versa* (second and fourth panels) than lead to a change in sense of rotation of helicopter type molecules (third panel). The observation that collisions where Δ*m*_*J*_ ≠ 0 occur signifies that the underlying interaction potential is not flat.^44^ As the temperature is increased two trends can be observed, an increase of state conserving cartwheel collisions (fifth panel) accompanied by a reduction of state conserving helicopter collisions (first panel).

We can also use the *S*-matrix elements to further investigate the stereodynamic effects involved in the specular scattering of H_2_ from the Cu(511) surface considered here. One informative characteristic that can be calculated is what we will denote as the rotational selectivity (RS) of the scattering channel. This corresponds to the relative probability of scattering into a particular channel for molecules which arrived at the surface as cartwheels or helicopters with respect to the surface normal (regardless of their final state after scattering). The RS for *m*_*J*_ = 1 and *m*_*J*_ = 0 is calculated by summing the square of the *S*-matrix elements over the columns corresponding to the initial *m*_*J*_ = 1 and *m*_*J*_ = 0 states and then taking the ratio using5
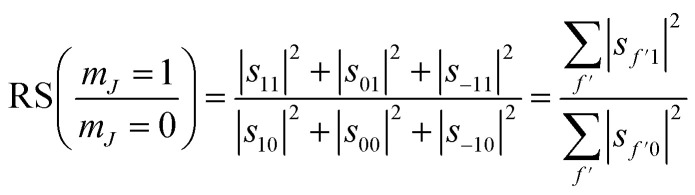


A second stereodynamic characteristic is the rotational polarisation (RP) of the scattering channel, which corresponds to the relative probability of a H_2_ molecule, which has scattered into a particular scattering channel, being in the *m*_*J*_ = 1 or *m*_*J*_ = 0 state after scattering with respect to the surface normal (regardless of its state before scattering). The RP for *m*_*J*_ = 1 and *m*_*J*_ = 0 is calculated by summing the square of the *S*-matrix elements over the rows and taking the ratio using6
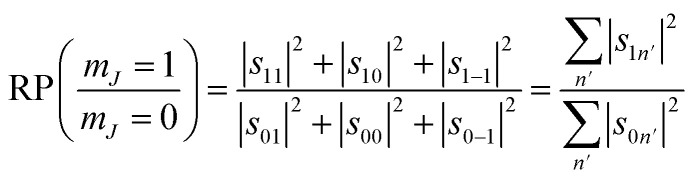


**Fig. 7:**
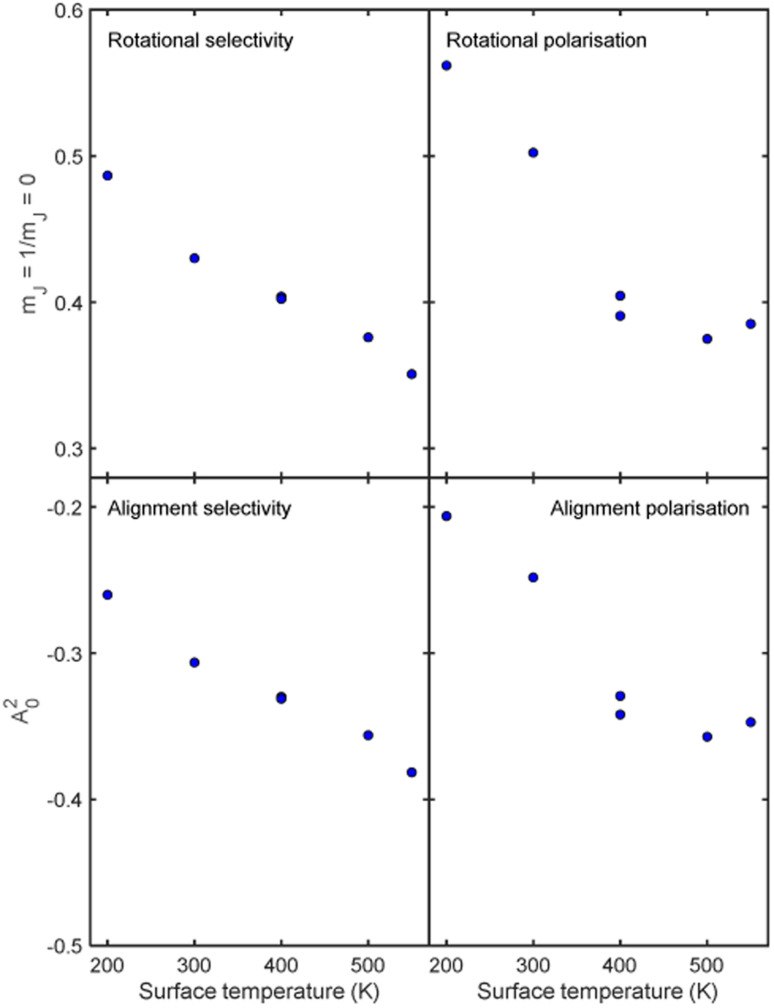
Top row. The rotational selectivity (left) and rotational polarisation (right) for *m*_*J*_ = 1 ‘helicopter’ and *m*_*J*_ = 0 ‘cartwheel’ rotating molecules for the best fits obtained for H_2_ scattering from the Cu(511) surface for each surface temperature. Bottom row. Alignment selectivity (left) and alignment polarisation (right) for the best fits obtained for H_2_ scattering from the Cu(511) surface for each surface temperature.

Whilst we have chosen to define these quantities with respect to the surface normal in the current work, it is important to emphasise that they can be defined with respect to any quantisation axis by rotating the scattering-matrix elements to that reference frame before applying eqn (5) and (6). The rotational selectivity and polarisation defined in eqn (5) and (6) are presented in the top left and top right panel of Fig. 7 respectively. There is a preference for H_2_ molecules that are rotating like cartwheels to be scattered into the specular channel, as indicated by the RS being less than one, a trend which increases even further as the surface temperature is increased. The RP is also less than one showing that the specular channel acts as a rotational polariser, *i.e.*, if the incident beam has no initial rotational polarisation, there will still be a bias towards more cartwheel molecules in the scattered beam. Similarly to the rotational selectivity, the rotational polarisation increases at higher surface temperatures. The observation that H_2_ molecules that are rotating like cartwheels are preferentially scattered into lower order diffraction peaks agrees qualitatively with earlier theoretical work on H_2_ scattering from a Co(0001) surface,^45^ which showed that cartwheeling molecules in lower |*m*_*J*_| states were more likely to be scattered into lower order diffraction peaks (including specular) than helicoptering molecules in higher |*m*_*J*_| states which either reacted or were scattered into higher order diffraction channels. This theoretical result was attributed to molecules with helicopter type rotation sampling a more corrugated potential.^45^

Another way to characterise the stereodynamics is through the alignment dependence of the scattering process, where the alignment parameter, *A*_0_^2^, is calculated as^8^7
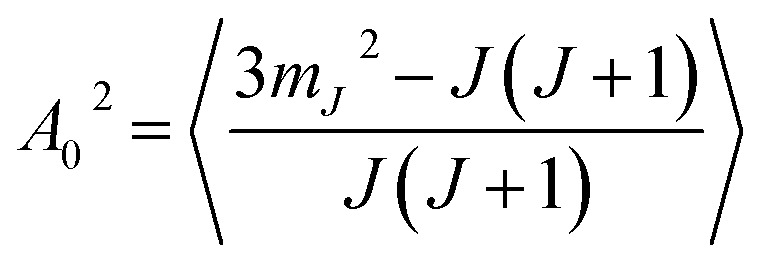


Analogously to the rotational selectivity considered above, we define an alignment selectivity (AS) which corresponds to the alignment of H_2_ molecules which scatter into a particular channel regardless of their state after the collision, which is found using8
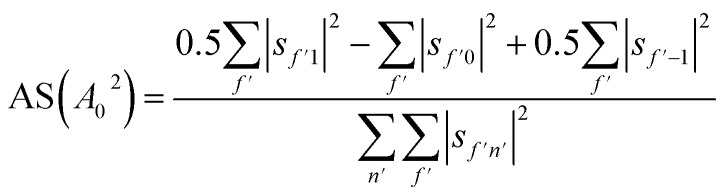
where the sums run over the 3 *m*_*J*_ states after scattering (*f*′). Likewise, we can quantify the alignment that the H_2_ molecules have after scattering into a particular channel regardless of their state before the collision through the alignment polarisation (AP) using9
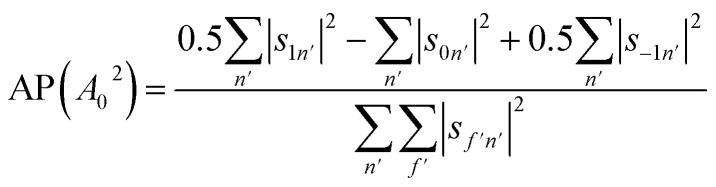
where the sums run over the 3 *m*_*J*_ states before scattering (*n*′). The values of the AS and AP parameters, which are shown in the bottom left and right panels of Fig. 7 respectively, mirror those obtained for the RS and RP shown in the equivalent panels in the top row of Fig. 7, with a value of *A*_0_^2^ = 0 corresponding to no alignment dependence and an increasingly negative value to a stronger preference for H_2_ molecules that are rotating like cartwheels (*m*_*J*_ = 0) to those that are rotating like helicopters (*m*_*J*_ = ±1). The magnitude of the alignment polarisation created by scattering an initially unpolarised beam of H_2_ into the specular channel from a Cu(511) is seen to increase as the surface temperature is increased. The rotational alignment of the scattered beam could therefore be tuned by changing the temperature of the Cu(511) surface.

Previous studies on rotational alignment effects in the interaction of H_2_ with copper surfaces have focussed on how this influences the dissociation probability, rather than the scattering probability, with there being several experimental^8,46,47^ and theoretical^17,48,49^ studies which have suggested that the dissociation of H_2_ in the vibrational ground state on copper surfaces is more probable for molecules that are rotating like a helicopter (*m*_*J*_ = ±1) than a cartwheel (*m*_*J*_ = 0). At higher surface temperatures, the reactivity also tends to increase.^5,50^ It could therefore be argued that as the surface temperature is increased more molecules which are rotating like helicopters will react which means that fewer will scatter, which could lead to the observation of there being more cartwheels scattered in general and into the specular channel in particular as the surface temperature increases. Whilst similar arguments have been used for equivalent observations in previous theoretical studies for the rotationally inelastic diffraction of H_2_ from surfaces,^51,52^ this is likely to be an over-simplified explanation in this case, due to the number of competing scattering (diffraction) channels, all of which will be affected by the changing surface temperature. Furthermore, whilst recording the data presented here we saw some evidence of H_2_ sticking on the Cu(511) surface which we used to obtain a very crude estimate of the sticking coefficient which was on the order of 10^−4^. Given that the absolute probability of scattering into the specular channel on the same surface is on the order of a few percent (*i.e.*, 10^−2^), it is unlikely that the surface temperature dependent observations reported here can be attributed just to the competition between sticking and specular scattering, meaning that the scattering into the (many) other diffraction channels and/or inelastic scattering plays the dominant role. It is also interesting to note that theoretical studies of other surfaces have predicted that the alignment dependence of the reactivity should decrease with increasing surface temperature,^14–17^ as the thermal motion of the surface atoms distorts the surface, reducing the activation barrier and allowing molecules in less favourable (cartwheel) geometries to react. This would be expected to decrease the polarisation of the scattered beam if these were the only two channels available which is the opposite to what we see in the present study.

Another possible mechanism for the increased rotational selectivity at higher surface temperatures could be due to H_2_ molecules that are rotating like helicopters being more sensitive to the changes in the Cu(511) surface structure as the temperature is increased as their plane of rotation is parallel to the surface, than cartwheels where the plane of rotation is perpendicular to it. Previous work has shown that the motion of sodium adatoms on a Cu(511) surface occurs mostly parallel to the step edges within the surface plane.^53^ Assuming that copper adatoms diffuse across the surface in a similar way, the interaction potential that the molecules that are rotating like helicopters sample is likely to be more affected by surface diffusion than those that are rotating like cartwheels. As scattering into the specular channel is a coherent phenomenon and is therefore sensitive to the long-range order of the surface, it could be that scattering of molecules that are rotating like helicopters becomes more incoherent at a faster rate as the surface temperature is increased than those that are rotating like cartwheels, as the helicopters are more sensitive to the disorder within the surface plane. This would lead to the decreasing helicopter to cartwheel rotational selectivity ratio and alignment selectivity as the surface temperature is increased, as shown in the first column of Fig. 7. Whilst this provides a possible alternative picture, the reality is again likely to be significantly more complicated, due to the many other elastic and inelastic channels that the H_2_ molecules can scatter into, which could favour scattering of molecules that are rotating like helicopters over those that are rotating like cartwheels as the surface temperature is increased.

## 5 Summary

The current study has investigated the stereodynamic effects for the specular scattering of H_2_ from a Cu(511) surface and how these change as a function of surface temperature. We have shown that the scattering channel is rotationally selective, with molecules rotating like cartwheels (*m*_*J*_ = 0) preferentially scattered into the channel over those that are rotating like helicopters (*m*_*J*_ = ±1), and likewise rotationally polarises the scattered beam, again with the molecules more likely to be rotating like cartwheels after the collision than helicopters. This polarisation dependence becomes stronger as the surface temperature is increased, with scattering from the Cu(511) surface becoming more selective for molecules in *m*_*J*_ = 0 at higher temperatures. In contrast, scattering from Cu(111) exhibits a rotational orientation dependence which does not vary with surface temperature over the range studied here. Scattering an initially unpolarised H_2_ beam from a Cu(511) surface will create a H_2_ beam which has a rotational alignment, where the polarisation of the beam can be tuned by changing the surface temperature. It is also interesting to note that the polarisation of the specularly scattered beam is opposite to that observed in our previous study of H_2_ scattering from LiF(100),^13^ where molecules in *m*_*J*_ = 1 were more likely to scatter into two different diffraction channels than those in *m*_*J*_ = 0.

The incorporation of surface temperature effects into theoretical models is an active and on-going area of research, with many studies focusing on the scattering of H_2_ from copper surfaces.^54–57^ Being able to follow the coherent evolution of the wave function during the collision of the molecule with the surface, and consequently determine how the scattering-matrix changes as a function of surface temperature makes the results presented here particularly valuable as a benchmark for testing and developing accurate theoretical models which incorporate the effect of surface temperature.

## Conflicts of interest

There are no conflicts to declare.

## Supplementary Material

FD-251-D4FD00007B-s001
