# Supplementary material for: Temperature dependent stereodynamics in surface scattering measured through subtle changes in the molecular wave function
Source: Faraday Discuss. 2024 Feb 14;251:76–91. doi: 10.1039/d4fd00007b (PMC11349063; doi:10.1039/d4fd00007b)
Supplement: FD-251-D4FD00007B-s001 [file FD-251-D4FD00007B-s001.pdf]

Electronic Supplementary Material (ESI) for Faraday Discussions.
This journal is © The Royal Society of Chemistry 2024

Supplementary Information for

Temperature dependent stereodynamics in surface scattering measured through subtle changes in the molecular wave function.

Helen Chadwick and Gil Alexandrowicz

In a previous publication we have shown using numerical simulations that the scattering-matrix parameters returned by the best fit successfully reproduces the true matrix values even in the presence of experimental noise[1]. In this Supplementary Information, we show that even if we take into account more results from fits with slightly larger deviations from the experimental data, all the stereodynamic trends reported in the main text are reproduced. In contrast, including all the fits, including those which deviate from the data in a way which is significant with respect to the experimental errors, leads to a large scatter of the scattering-matrix parameters.

Scattering-matrix elements and scattering probabilities

The scattering-matrix amplitudes and phases obtained from the best 20 fits (coloured bars) and all 100 fits (white bars) are shown in Fig. S1 and Fig. S2 respectively for H_2 scattering from Cu(511) at surface temperatures of 200K (first row), 300K (second row), 400K (third row), the 400K repeat measurement (fourth row), 500K (fifth row) and 550K (sixth row). The results from all the fits cluster around the results from the best 20 fits, which are taken to be those with the lowest error between the calculated and measured oscillation curves.

In the case of the scattering-matrix amplitudes, all the histogram distributions contain fits where the amplitude is effectively 0, and in some cases this can be a relatively large number of fits giving rise to a double peak in the histogram. For example, in the second panel of the top row in Fig. S1, which corresponds to the s_{10} (and by symmetry s_{-10}) amplitude at a temperature of 200K, over 10 out of the 100 fits give a value of 0 for this parameter. In Fig. S3, we compare the fit which gives the lowest error over all (red dashed line) and the fit with the lowest error which has s_{10} = 0 (blue dashed line), which clearly shows that this gives an inferior fit to the measured signals, deviating from the signal in a way which is significant with respect to the experimental error bars, and therefore can, justifiably, not be included in the analysis of the stereodynamic effects reported in the main manuscript. To provide some context for the following discussion, this corresponds to the fit with the 48th lowest error.

Whilst only the fits which produced the lowest error were used to produce the plots in the main manuscript, Fig. S4 and Fig. S5 present the scattering-matrix elements and m_J state to m_J state scattering probabilities averaged over the best 20 fits (blue), 40 fits (black) and all 100 fits (red) respectively. Using the average scattering-matrix elements as considered here is likely to not produce a signal which matches the experimental data when they vary significantly between fits as the signal is an interference phenomenon, and therefore changing amplitudes and phases even by a small amount can change the calculated signal significantly. Despite this, it is useful to get an idea of the uncertainty that the parameters that we report have and how much they vary between the fits. Whilst in most cases the differences between the analysis with 20 fits (blue) and 40 fits (black) is extremely small, there are some which are larger, for example in the s_{10} (first row, second panel, Fig. S4) and s_{00} (first row, fifth panel, Fig. S4) amplitudes where the two repeat measurements performed at 400K give visible differences between the parameters (although these are still small). This leads to the differences seen in the state-to-state scattering probabilities for the same transitions in Fig. S5. Further increasing the number of fits to include all the fits has a more significant effect on the parameters, although this now also includes complete misfits to the data.

Polarisation properties

The polarisation properties considered in the manuscript are presented in the same way for the different surface temperatures for scattering from Cu(511) in Fig. S6. Again, the fits with higher errors (white bars) still tend to cluster around the results from the best 20 fits (coloured bars). Figure S7 presents the data for the rotational selectivity (first panel) and rotational polarisation (second panel) for m_J = 1 and m_J = 0, and the alignment selectivity (third panel) and alignment polarisation (fourth panel) as a function of surface temperature averaged over the best 20 fits (blue), 40 fits (black) and 100 fits (red). In the case of 100 fits in the left two panels some of the points are not visible, as some of the fits with larger errors

have very small $m_J = 0$ values, which makes the ratio several orders of magnitude larger than the y-axis scale, which has been kept similar to the other figures in the paper and Supplementary Information for ease of comparison. Comparing the black and blue points, the values that quantify the polarisation properties presented in the main manuscript are not significantly different whether the best 20 or 40 fits are analysed for Cu(511), demonstrating that the conclusions we reach on the temperature dependence of the stereodynamic effects presented using only the best fit in the manuscript are robust.

References

1 Y. Alkoby, H. Chadwick, O. Godsi, H. Labiad, M. Bergin, J. T. Cantin, I. Litvin, T. Maniv and G. Alexandrowicz, *Nat. Commun.*, 2020, **11**, 3110.

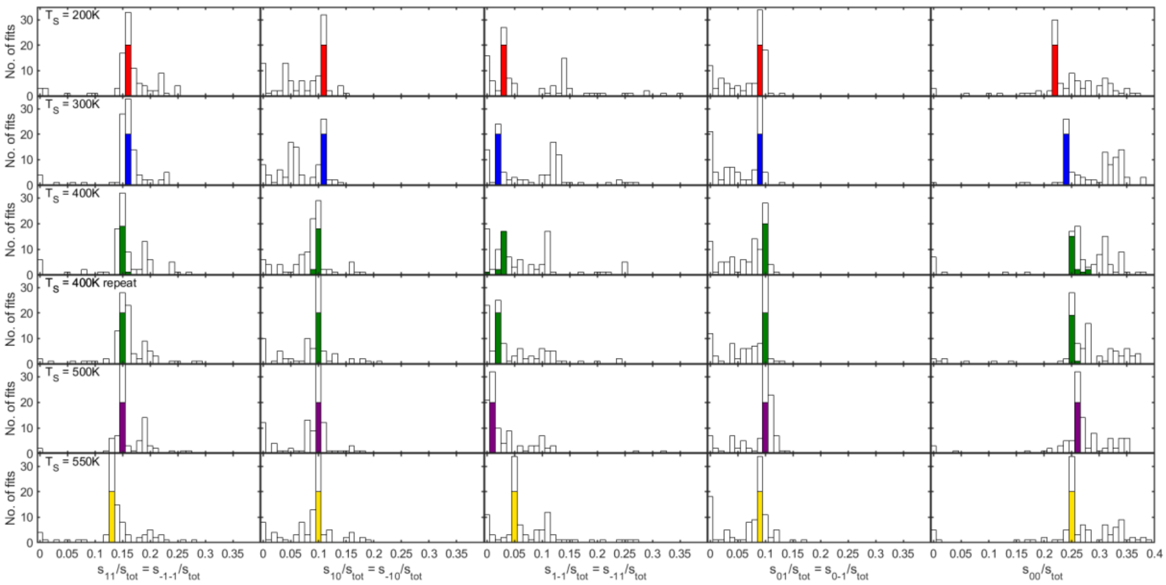

Figure S1. Histograms of the scattering-matrix amplitudes normalised to the total scattering-matrix amplitude for $s_{11} = s_{-1-1}$ (first column), $s_{10} = s_{-10}$ (second column), $s_{1-1} = s_{-11}$ (third column), $s_{01} = s_{0-1}$ (fourth column) and s_{00} (fifth column) obtained from the 20 best fits (those with the lowest error, coloured bars), and from all 100 fits (white bars) for H_2 scattering from Cu(511) at a surface temperature of 200K (first row), 300K (second row), 400K (third row), the repeat 400K measurement (fourth row), 500K (fifth row) and 550K (sixth row).

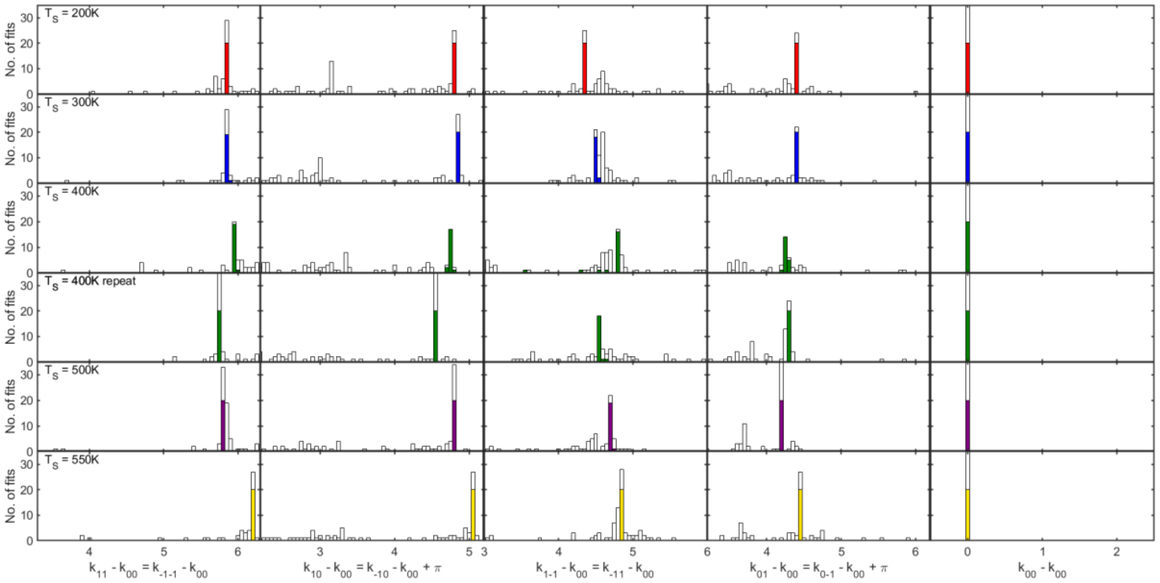

Figure S2. Histograms of the scattering-matrix phases with respect to k_{00} for k_{11} = k_{-1-1} (first column), k_{10} = k_{-10} + π (second column), k_{1-1} = k_{-11} (third column), k_{01} = k_{0-1} + π (fourth column) and k_{00} (fifth column) obtained from the 20 best fits (those with the lowest error, coloured bars), and from all 100 fits (white bars) for H_2 scattering from Cu(511) at a surface temperature of 200K (first row), 300K (second row), 400K (third row), the repeat 400K measurement (fourth row), 500K (fifth row) and 550K (sixth row).

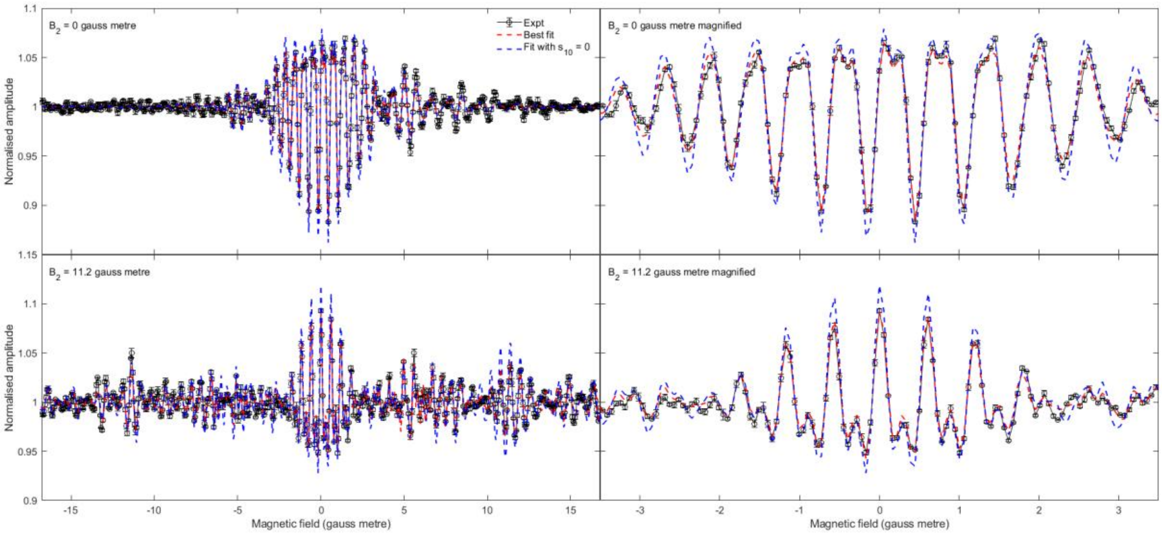

Figure S3: The best fit (dashed red line) and a fit where one of the scattering-matrix amplitudes (s_{10}) is 0 (dashed blue line, see text for details) to the oscillation curves measured (black) for the specular scattering of H_2 from Cu(511) at a surface temperature of 200K for B_2 = 0 gauss metre (first row) and B_2 = 11.2 gauss metre (second row) for the full measurement range (first column) and a magnification of the central oscillations (second column).

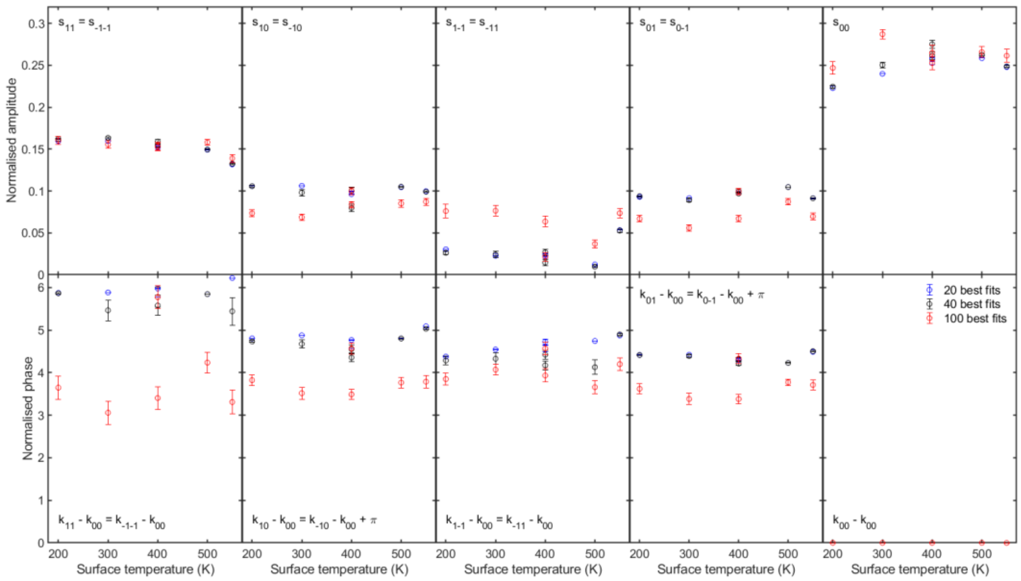

Figure S4. The average S-matrix amplitudes (first row) and average phases (second row) obtained from the best 20 fits (blue), 40 fits (black) and 100 fits (red) for H_2 scattering from Cu(511) as a function of surface temperature. The scattering-matrix parameter each plot corresponds to is given in each panel.

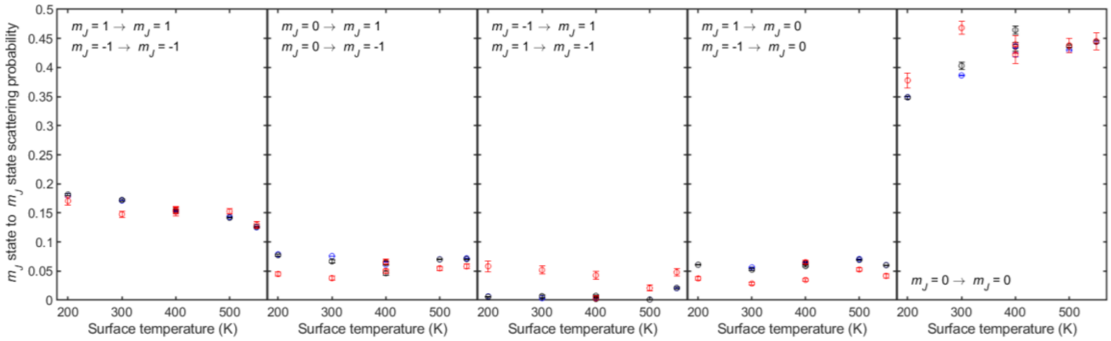

Figure S5. The average m_J state to m_J state scattering probabilities obtained from the best 20 fits (blue), 40 fits (black) and 100 fits (red) for H_2 scattering from Cu(511) as a function of surface temperature. The initial and final m_J state shown is given in the text of each panel.

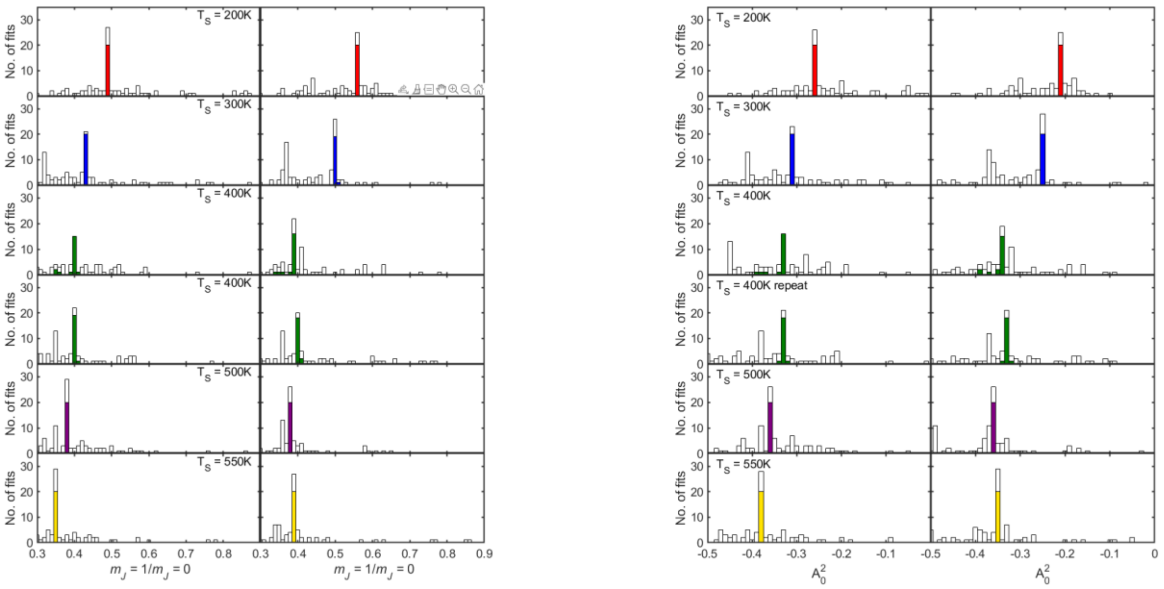

Figure S6. The rotational selectivity (first column) and rotational polarisation (second column) for the m_J = 1 'helicopter' and m_J = 0 'cartwheel' states, and alignment selectivity (third column) and alignment polarisation (fourth column) obtained from the 20 best fits (those with the lowest error, coloured bars), and from all 100 fits (white bars) for H_2 scattering from Cu(511) at a surface temperature of 200K (first row), 300K (second row), 400K (third row), the repeat 400K measurement (fourth row), 500K (fifth row) and 550K (sixth row).

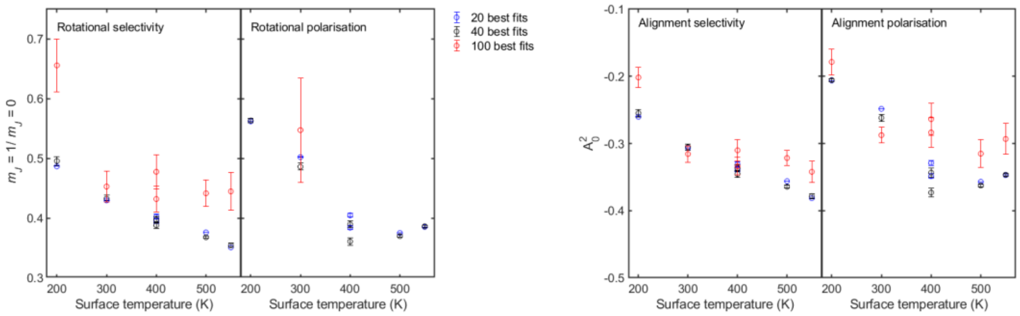

Figure S7. The average rotational selectivity (first panel) and average rotational polarisation (second panel) for the m_J = 1 'helicopter' and m_J = 0 'cartwheel' states, and alignment selectivity (third panel) and alignment polarisation (fourth panel) obtained from the best 20 fits (blue), 40 fits (black) and 100 fits (red) for H_2 scattering from Cu(511). The rotational polarisation for 100 fits in the second panel give values significantly out of the range of the y-axis and span several orders of magnitude.
